# Supplementary material for: Fentanyl Induces Novel Conditioned Place Preference in Adult Zebrafish, Disrupts Neurotransmitter Homeostasis, and Triggers Behavioral Changes
Source: Int J Environ Res Public Health. 2022 Oct 19;19(20):13533. doi: 10.3390/ijerph192013533 (PMC9603063; doi:10.3390/ijerph192013533)
Supplement: Supplementary file 1 [file ijerph-19-13533-s001.zip › ijerph-1923001-supplementary.pdf]

Fentanyl induces novel conditioned place preference in adult zebrafish, disrupts neurotransmitter homeostasis, and triggers behavioral changes

Yuanzhao Wu[1], Anli Wang[2], Lixiang Fu[1,3], Meng Liu[1], Kang Li[1], Song Chian[1], Weixuan Yao[1], Binjie Wang[1,*]， Jiye Wang[1, †]

Email: wangbinjie@zjpc.edu.cn

This PDF file includes:
Supplementary Methods
Tables S1

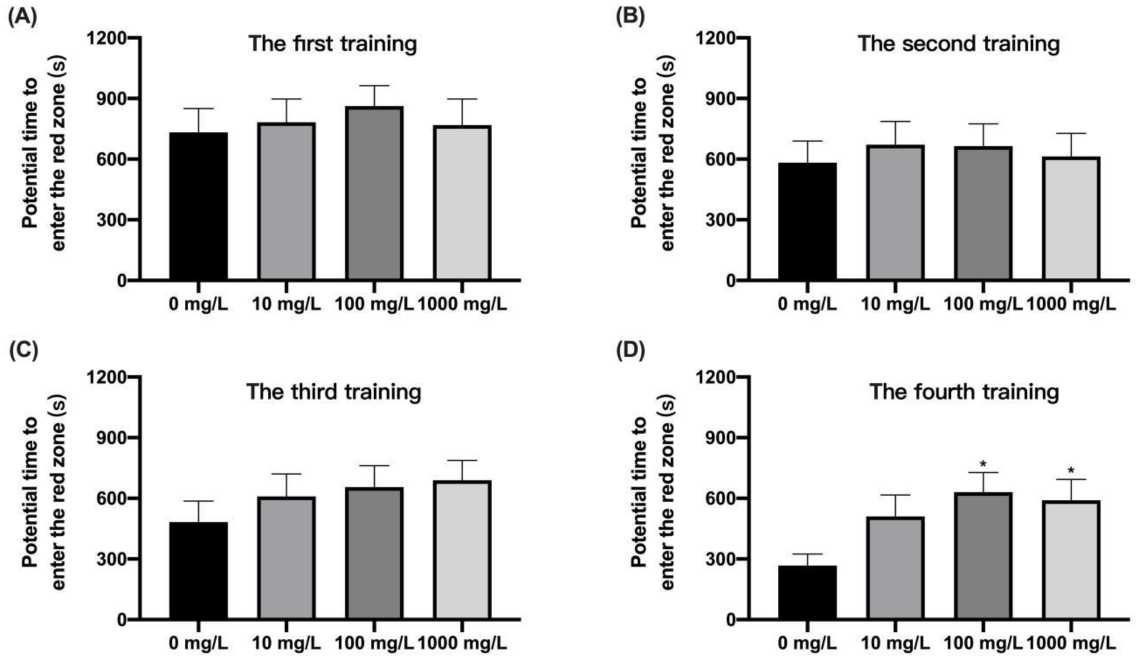

Figure S1. The potential time for zebrafish to reach the red area for the first time in four T-maze training sessions. (A-D) Data from four training sessions. Data were expressed as mean ± SEM, all behavioral parameter data were analyzed by one-way ANOVA followed by Dunnett's T3 multiple comparisons test. The level of significance was defined as *$p < 0.05$.

Methods：

LC-MS/MS analysis was performed using an LCMS 8050 ultra performance liquid chromatograph-mass spectrometer with an electron spray ionization source (ESI) and a triple quadrupole mass analyzer (Shimadzu, Japan) and a TURBOVAP II nitrogen blowing concentrator (Biotage, Sweden). An ACQUITY UPLC HSS T3 (2.1 mm×100 mm×1.8 μm) was used as the liquid chromatographic column. The mobile phase A was water (0.1% formic acid) and the mobile phase B was methanol at a flow rate of 0.35 mL/min. The LC gradient used for chromatographic separation started at 100% A and decreased to 90% B in 0.8 min. From 0.8 to 1.1 min, the A phase decreased to 10% and was maintained for 0.6 min. From 1.6 to 2.5 min, the A phase decreased to 5% and was maintained for 1.0 min. Finally, phase A returns to 100% and is rinsed for 1 min. Electrospray source ESI (positive ion mode); nebulizing gas: nitrogen, flow rate 3 L/min; drying gas: nitrogen, flow rate 10 L/min; collision gas: argon, DL temperature 250 °C; interface temperature 300 °C, heating block temperature 400 °C; scan mode: multiple reaction monitoring mode (MRM), segmented acquisition. The strongest fragment as the quantitative ion and the most intense fragment as the quantitative ion and the second strongest fragment as the qualitative ion.   Neurotransmitter concentration detection was

performed using an external standard method. According to the above conditions to optimize the mass spectrometry parameters, the qualitative and quantitative ion pairs and other information of the five neurotransmitters were shown in Table S1.

Supplementary Table S1. Analysis parameters of five neurotransmitters. Ions with * were quantitative ions.

Number	Compound	Retention time （min）	Precursor ion （m/z）	Product ion （m/z）	Q1 Pre deviation （V）	Collision voltage （V）	Q3 Pre deviation （V）
1	Gln	0.88	147.0	84.2*; 130.2	-18; -18	-18; -14	-17; -14
2	GABA	0.89	104.2	87.1*; 45.1	-20; -12	-13; -22	-17; -16
3	DA	1.51	154.0	137.0*; 91.1	-20; -12	-13; -24	-15; -19
4	5-HT	2.26	177.1	160.2*; 115.2	-12; -13	-13; -28	-17; -12
5	cortisol	3.34	363.2	331.2*; 107.0	-13; -13	-11; -39	-12; -11
